# A synthesis of implementation science frameworks and application to global health gaps

**DOI:** 10.1186/s41256-019-0115-1

**Published:** 2019-08-27

**Authors:** Pablo Villalobos Dintrans, Thomas J. Bossert, Jim Sherry, Margaret E. Kruk

**Affiliations:** 1 Harvard T H Chan School of Public Health, Boston, USA; 2 Universidad de Santiago, Santiago, Chile; 3 University Research Co., LLC, Chevy Chase, USA

**Keywords:** Implementation science frameworks, Global health gaps

## Abstract

**Background:**

Implementation science has been growing as discipline in the past decades, producing an increasing number of models in the area. On the other hand, most frameworks are intended to guide the implementation of programs, focusing on identifying elements and stages that increase their success. This article aims to structure this discussion, proposing a simplified tool that synthesizes common elements of other frameworks, and highlight the usefulness to use implementation science not only in identifying successful implementation strategies but as a tool to assess gaps in global health initiatives.

**Methods:**

The study was carried out through a combined methodology that included an initial search of implementation science frameworks, experts’ opinions, and the use of references in frameworks to elaborate a list of articles to be reviewed. A total of 52 articles were analyzed, identifying their definitions of implementation science and the elements of different frameworks.

**Results:**

The analysis of articles allowed identifying the main goals and definitions of implementation science. In a second stage, frameworks were classified into “time-based”, “component-based” and “mixed”, and common elements of each type of model were used to propose a synthetic framework with six elements: Diagnosis, Intervention provider/ system, Intervention, Recipient, Environment, and Evaluation. Finally, this simplified framework was used to identify gaps in global health was using The Lancet Global Health Series. Potential areas of intervention arise for five different global health issues: malaria, non-communicable diseases, maternal and child health, HIV/AIDS, and tuberculosis. Prioritization strategies differ for the different health issues, and the proposed framework can help identify and classify all these different proposals.

**Conclusions:**

There is a huge variety of definitions and models in implementation science. The analysis showed the usefulness of applying an implementation science approach to identify and prioritize gaps in implementation strategies in global health.

**Electronic supplementary material:**

The online version of this article (10.1186/s41256-019-0115-1) contains supplementary material, which is available to authorized users.

## Introduction

During the past decades, implementation science (IS) has been recognized as increasingly important. Practitioners and scholars have realized the difference between efficacy (outcome of an intervention under ideal conditions) and effectiveness (outcome of an intervention under normal conditions) when translating evidence-based research into practice in the real world [1–4].

The growing importance of the field has resulted in a huge increase of research in the area; a search for “implementation science” in PubMed shows that the number of articles on the topic has increased from 141 in 2000 to more than 2500 in 2015; since 2004, at least ten journals have devoted special issues to the topic [5].

The proliferation of studies has assisted scholars and practitioners by providing more guidance on concept and design [5], but the proliferation of definition, models, and methods has created new complexities for users [6–8]. It is challenging to reconcile several of the popular frameworks, which approach implementation science from diverse perspectives. [9]. The breadth of the field can be a barrier to use of implementation science, particularly for practitioners and policy makers approaching the area for the first time. [5, 10–14] As a result there have been several efforts to compile and standardize terms and definitions [7, 15–18] as well as to identify and classify the plethora of frameworks and models that have been proposed [5–7, 19–25].

From an academic perspective, the diversification of the conceptual underpinnings in the field may hinder robust interdisciplinary dialogue and increases the risk of “interdisciplinary amateurism”, i.e. an exchange of ideas without grasping their full implications [26].

Finally, implementation science frameworks and models have been presented as useful in recognizing elements for successful implementation, but could also be valuable in identifying current gaps in implementation strategies as well as helpful for prioritizing areas for action.

The aim of this paper is to compile and classify key implementation science frameworks in health care and synthesize their key features. We apply the synthesis of frameworks to identify core challenges in the implementation of priority programs in global health.

## Methods

### Search and analysis strategies

**Fig. 1:**
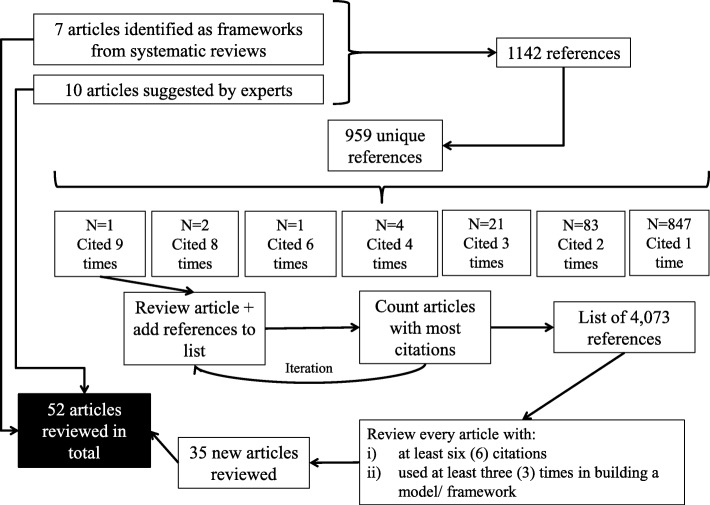
Flowchart of the search and selection process. Source: Authors elaboration.

We set out to identify the main definitions and aims of implementation science and commonly used frameworks and models. The search process was performed in three stages. First, we began by reviewing existing frameworks on implementation science. In the first stage, we used a targeted review, searching in PubMed for papers in English published in peer-reviewed journals between 2000 and 2016. The terms used in the search were: “implementation science”, “implementation research”, “framework”, and “systematic review”. As highlighted by other authors [5, 22], the diversity of terms, definitions, and disciplines contributing to implementation science make it difficult to locate every publication in the field and carry out a traditional systematic review. The selection of articles was focused on publications containing models and frameworks for implementation science based on systematic reviews, exclusively. The initial search resulted in seven systematic review articles, which were examined in depth. A second set of ten papers was included based on suggestions from experts in the field; all the suggested articles not considered initially (although some were not systematic reviews) were included in the list of articles to be analyzed. Finally, the list was completed using the references in all articles included in the first two stages (snowball sampling). From the original list of references, the articles with more citations were identified, analyzed and their bibliography included in the list of references. From these references, new articles were selected and their references added to the list. After several iterations, 4073 references were identified; out of these the articles included in the final selection were those with six citations or more, as well as models explicitly used in building a new framework or model (with three citations or more). The selection process used to select articles in the review is shown in Fig. 1:

Even though there is an important overlap between both groups, the strategy allowed identifying articles that were relevant for the implementation science literature, even though they contained no formal model, and top reference models that were not among the top cited articles (see Additional file 1). The total number of articles reviewed was 52, comprising scientific articles and books presenting frameworks, meta-frameworks, theories, classifications, and concepts and definitions [2, 5–9, 13–25, 27–59].

All the articles were examined to identify the presence of a framework/ model of implementation science. Each paper was reviewed, extracting information on:

1. Definitions and goals of implementation science
2. Methods (whether it was based on a systematic search or not)
3. Elements of the framework
4. Focus of the framework
5. Context of implementation

A first by-product of the search and review is displayed in Additional file 1. It exhibits the list of articles included in the review, showing the most cited references as well as the most used frameworks. The table allows shows relevant references that can serve as a starting point for a practitioner or decision maker interesting in understand and use implementation science, identifying articles containing frameworks, classifications and those based on systematic searches.

## Results

### Definitions and aims of implementation science

We reviewed the frequency of words and phrases used in the definitions and stated goals of implementation science. Table 1 shows the main concepts used to define implementation science; 86 different definitions were extracted and synthesized using the structure shown in the table.

**Table 1:** Words used in different definitions of “implementation science”

From			Goal	
Action/ tool	Starting point	Destination	Action/ tool	Result
Translating/ transferring/ transporting/ exchanging (9)	Intervention/ program (20)	Practice/ routine practice (29)	Improve/ change (12)	Quality of health-care (11)
Reducing/ closing gap (8)	Research (11)	Use/ utilization/ routine use (14)	Meet/ achieve (3)	Intervention/ implementation (4)
Promoting uptake (8)	Knowledge (10)	Real-world/ reality/ context/ settings (9)	Deliver (2)	Health-care (3)
Getting/ bringing/ delivering (6)	Evidence (9)	Innovation/ program/ policy (4)	Identify/ clarify (2)	Outcomes/ health outcomes (2)
Understanding (4)	Practice (8)	Knowledge (3)	Other (5)	Other (6)
Implementing (4)	Innovation (5)	Other (10)		
Putting (4)	Science/ findings (4)			
Bridging/ nexus (5)	Other (8)			
Integrating (3)				
Promoting/ encouraging (3)				
Process (3)				
Other (10)				

Note: Parentheses indicate the number of times a concept was used in the structure of the definitions. Totals in every column differ since not every definition included all the elements displayed in the table

Most definitions followed the pattern described: an action to move from a starting to a final stage, and a goal. It is clear that implementation science relates to the idea of transferring evidence-based knowledge into practice, in a scientific way.

**Fig. 2:**
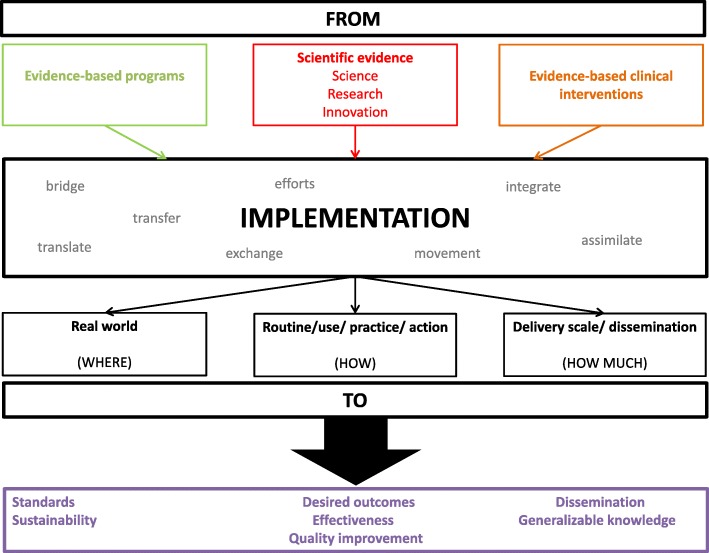
Goals of Implementation Science. Source: Authors elaboration.

As shown in Table 1, the different definitions were analyzed to identify the components of a definition -action, starting point and destination, and expected result- and the concepts and terms utilized for each component. This information was then used to build what can be seen as a generic definition of implementation science. Figure 2 presents a visual representation of the goals of implementation science synthesized from the reviewed literature. It is important, however, to recognize that the use of different terms is not whimsical: different definitions emphasize different dimensions of implementation science. For example, even though all the definitions follow the proposed scheme (from knowledge to practice) they differ on the starting point (basic science, innovations) and the outcome (applied in the real world, routine, scaling-up). As shown below, it starts from already known evidence about what works and tries to fill the gap between this knowledge and its application to a particular setting, reducing the gap between theory and practice. The final objective of this process is quality improvement and moving from efficacy-proven interventions to effective intervention in the real world.

We then developed a classification of the conceptual models used in implementation science. The first step in the process was to identify differences and common elements among existing models and grouped them into different groups, similarly to other studies that also classify models and frameworks [7]. Using the focus on the implementation process, the frameworks were divided into two broad categories: time-based and component-based, resembling Nilsen’s classification between “Process (how-to) model” and “Determinant framework” [19]. The list of papers reviewed and their categories can be found in the Additional file 2.

The set of papers used includes several systematic reviews as well as non-systematic ones, mostly based on previous studies (empirical and non-empirical) written in English and including one or more framework in implementation science or implementation processes, covering a period of more than 45 years. Since the search was restricted to PubMed, most of the frameworks are built within the scope of health care innovations, but some claim to be generalizable to other areas outside health care [16, 28, 33] or constructed to analyze particular diseases or populations [7, 17, 18, 28, 55].

Time-based models define the elements as a sequence of stages to be followed in order to have an adequate process of implementation, while component-based models focus on the presence of certain features that should be taken into account and planned for a successful implementation. There is an overlap between both types of frameworks, but also idiosyncratic parts that are missed from one and other. As noted before, given its complexity and specificity, implementation science frameworks and models tend to be incomplete [6].

Every framework was labeled using these two broad categories; in this process, it became clear that some frameworks included both approaches, allowing for a third category of “mixed frameworks”, in which elements of component-based and time-based are present. Once the groups were defined, the common components of each category were identified (Table 2).

**Table 2:** Classification of implementation science conceptual frameworks

Time- based frameworks		
*Basic question*	*Meta-stages*	*Stages*
When?	Pre-Implementation	Diagnosis
		Planning
	Implementation	Acting
		Monitoring
	Post-Implementation	Evaluation
		Changes
Component-based frameworks		
*Basic question*	*Meta-units*	*Units*
Who?	People	Provider
		Recipient
What?	Element	Intervention
		Environment

Time-based models highlight the *when*. Three meta-stages were identified from the different frameworks: pre-implementation, implementation, and post-implementation. The meta-stage of *pre-implementation* can be subdivided into smaller stages: *diagnosis* and *planning*. Some elements (stages) included in these phases are “set-up”, “research/ gather evidence”, “creating structures”, “develop a concrete proposal/ plan”, etc. The *implementation* meta-stage can be divided into the stages of *acting* and *monitoring*. Examples included in the original frameworks for these categories are “do”, “program installation”, “carry out plan and evaluate progress”, or “go full scale”. Finally, the *post-implementation* includes the process of *evaluation* and making *changes*. Several frameworks include in this meta-stage actions as “evaluate”, “test scale-up”, or “improve future applications”.

Component-based models focus on two different questions: *who* and *what*. These questions give rise to two different meta-units: people and elements. When focusing on people, different frameworks highlight the importance of considering characteristics of both, *providers* and *recipients*. Features related to the former are “inner-setting”, “organization”, “facilitation”, or “factors and support systems”. From the recipients’ point of view, several models emphasize the importance of elements such as “outer-setting”, “patient”, “community level factors”, “adoption/ assimilation”, or “participant’s responsiveness”. The meta-unit of elements is separated between elements present in the *intervention* itself, as well as elements to consider related to the *environment*. The frameworks studied identify “intervention/ innovation characteristics”, “program”, or “adherence/ intervention complexity” as components related to the intervention itself. Other components such as “outer-setting”, “structural factors”, “context”, or “external environment” can be defined as related to the environment.

### A synthesis of frameworks for implementation science

**Fig. 3:**
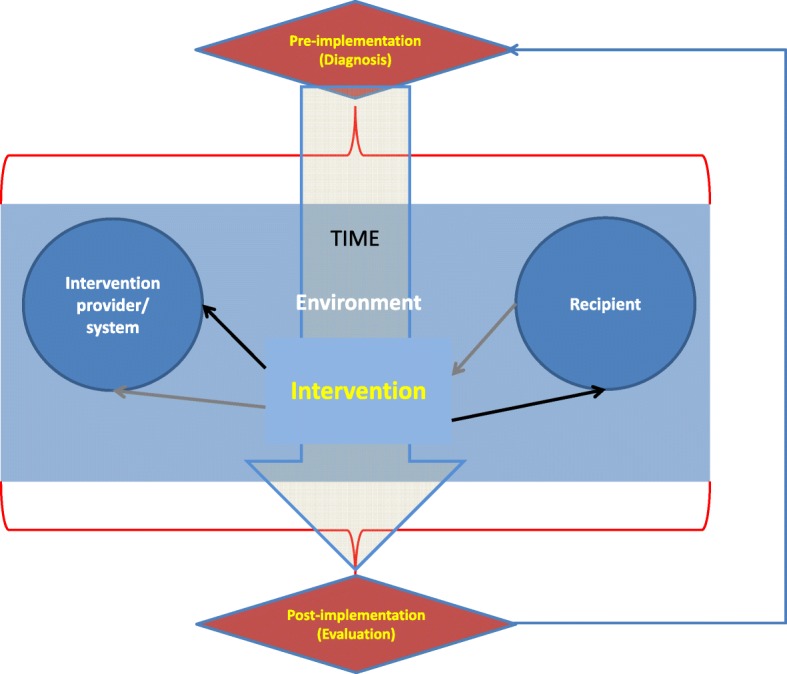
A synthesis of implementation science frameworks. Source: Authors elaboration

We synthesized the key features of existing frameworks. As shown in Fig. 3, the resulting framework contains six elements and synthesizes the frameworks previously presented: Pre-implementation (diagnosis), Intervention provider/ system, Intervention, Recipient, Environment, and Post-implementation (evaluation).

To address the apparent lack of dialogue between the two types of models, the three meta-stages of the time-based models were preserved, including all the parts identified as relevant in component-based models. Essentially, all the components of these models are referred to the implementation meta-stage. The framework’s synthesis adds a temporal dimension to the component-based models, as well as important new elements to be considered in the diagnosis and evaluation stages (environmental factors). Alternatively, the synthesis can be interpreted as an extension to time-based models, enhancing the importance of the implementation stage by defining concrete components to be assessed when implementing an intervention.

Figure 3 should be interpreted as a synthesis of both kinds of models: it not only adds elements of both types of frameworks, but also delineates their interaction. The time component is illustrated vertically in the figure. The process starts with the *Pre-implementation (Diagnosis)* phase, which explicitly takes into account every element of the implementation process: the initial assessment and the planning of the implementation should consider characteristics of providers and recipients, as well as environmental factors to design an effective intervention. Similarly, the *Post-implementation (Evaluation)* process also needs to consider all these factors when assessing performance and proposing changes. The use of implementation elements as input for the pre and post-implementation stages is illustrated by the red curly brackets in the figure.

The central box describes the *Implementation* stage, including: i) *Intervention provider/system*, i.e. the agent or system in charge of executing the intervention; ii) *Recipient*, i.e. the agent who is intended to be changed by the intervention; iii) the *Intervention* itself and; iv) *Environment*, i.e. all the external factors that affects the result of the intervention during the implementation stage. Providers, recipients and interventions are considered as independent components and illustrated in individual boxes, while environmental factors can affect any of these elements during the implementation process and, therefore are unbounded in this central box. Even though they are considered independent elements, providers, recipients and intervention relate to each other during this stage. The black arrows show the direction in which the provider affects the recipient *through* the intervention; on the other hand, the gray arrows denote the fact that the intervention and its results can affect providers (and consequently may lead them to change the activity) during the implementation process, a feedback usually related to the results of monitoring.

The three time-stages are connected in a sequential way, starting from the diagnosis and ending with the evaluation. However, an arrow connects the evaluation with the diagnosis, adding a cyclical component to the temporal dimension. Since both process (diagnosis and evaluation) are based in the assessment of the same elements (those included in the implementation phase) a natural process of feedback arise from the evaluation’s results which can be considered a new diagnosis in the process of changing the intervention.

Finally, original frameworks were “translated” into the new proposed framework: all the elements originally listed in each framework were reclassified into the six proposed categories, in order to ensure the completeness of the new model.

### Application of the synthesis to implementation of priority global health programs

Finally, the pertinence of the proposed synthesis was tested using several priority global health issues. It is designed to be, at the same time, sufficiently comprehensive so as to identify gaps and key elements needed when implementing programs, and simple enough to serve as guide for practitioners.

Five global health issues were identified and assessed using the synthesis of frameworks to organize and identify gaps and key elements of the implementation process. Malaria, maternal and child health (M&CH), non-communicable diseases (NCDs), HIV/ AIDS and tuberculosis (TB) were selected as current relevant global health issues.

Strategies to cope with these problems were collected using articles published in The Lancet Global Health Series. The use of Lancet series serves as an example to illustrate the utility of the synthesis for analyzing topics related to the Millennium Development Goals (MDGs) as well as new health conditions.

Several papers were selected through critical review process, using as criterion whether they identified gaps or contained proposal to deal with unfinished problems related to these global health issues [60–72]. All articles were reviewed to identify concrete strategies proposed to deal with the selected health conditions. These strategies were classified into the six categories described above: Diagnosis, Provider, Intervention, Recipient, Environment, and Evaluation. In a second stage, these strategies were regrouped and used to establish 23 sub-categories within the original classifications, and each one of the strategies identified was classified into a sub-category (Table 3).

**Table 3:** Categories, sub-categories and examples from the Lancet series

Category	Sub-categories	Examples	Malaria	NCDs	Maternal & child care	HIV/ AIDS	TB
DIAGNOSIS	Understanding disease and solution (intervention effectiveness)	Malaria: Resistance to drugs M&CH: Effectiveness and cost-effectiveness of nutritional interventions	L		L	H	H
	Population's health status: burden of disease/ risk factors	NCDs: Estimate NCD burden of disease and risk factors HIV/ AIDS: Need and context of specific populations		H	L	L	H
	Context/ environment: External factors	Malaria: Consider situation of neighbors M&CH: Infectious diseases, helminths, environmental enteropathy	L		H		
	Context/ environment: Internal factors	NCDs: Develop a national plan HIV/ AIDS: Include people living with HIV in the decision-making process in policy design		H	H	H	
	Financing	M&CH: Financial effects TB: Financing (cost of treatment)			H		H
PROVIDER	Workers' capacity (training)	NCDs: Strengthen capacity for change: human M&CH: Workforce planning and upgrade specific skills	H	H	H		
	System's capacity	TB: Integrated health care HIV/ AIDS: Operational convergence of HIV service-delivery platforms and other health issues			H	H	L
	Protocols and guidelines	HIV/ AIDS: Clear treatment guidelines TB: Standardization of treatment			H	H	H
	Financing	NCDs: Strengthen capacity for change: financial M&CH: Financing		H	H		H
INTERVENTION	Detection/ prevention	TB: New diagnostics (improving detection) NCDs: Prevention and treatment programmes	H	H		L	L
	Use of effective treatment	Malaria: Use effective treatment (primaquine) TB: Use of effective, tried and tested interventions	H	H	L	H	L
	Monitoring	NCDs: Monitoring M&CH: Monitoring coverage levels	H	H	H	H	
	Scale/ coverage	M&CH: Equity in provision HIV/ AIDS: Design targeted interventions (no one-size-fits-all approach)			L	L	L
RECIPIENT	Characteristics of the treated (individuals)	TB: Patient characteristics and the nature of their demands Malaria: Mobile vs. non-mobile population	H		L	H	L
	Characteristics of the untreated (community)	NCDs: Promote civil engagement M&CH: Community empowerment, advocacy and engagement	H	H	L		H
ENVIRONMENT	External support/context (international community)	Malaria: Support from international institution TB: Technical and financial support from countries	H		H	H	H
	Internal support/ context (politics and society)	NCDs: Political leadership HIV/ ADIS: Reform of justice systems	L	L	L	L	
	Internal conditions	TB: Overcrowding, indoor air pollution M&CH: Food prices			L		L
	Health system	M&CH: Health insurance and infrastructure TB: Integrated health care	H		L		H
	Financing	HIV/ AIDS: Investment in R&D Malaria: Funding			L	H	L
EVALUATION	Evaluation system	TB: Monitoring and assessment Malaria: Monitoring systems (surveillance): measurement of progress	H		L		
	Use of information	NCDs: Accountability: Review, assess and report progress M&CH: Quality improvement mechanisms in health facilities		H	H		
	Data availability	M&CH: Quantity and quality of monitoring data			H		
Total number of strategies proposed in the series			18	11	87	25	41
Median number of strategies for each sub-category			1	1	3.5	2	1.5

The table presents examples found for each sub-category, as well as a heat map reflecting the relative importance of each subcategory for every global health issue. Letters in each box represent the number of strategies or gaps mentioned by the articles in each health issue: H (high) for subcategories with a number of strategies above the median, L (low) for subcategories with a number of strategies below the median, and blank for subcategories with no strategies proposed. First, from an implementation science perspective, it is important to highlight that every subcategory is relevant for implementing strategies for change: blank boxes represent areas that were not prioritized in the reviewed articles but need to be pondered when designing, implementing and evaluating global health interventions. Boxes with a large number of strategies (H) identify areas considered as key in the reviewed articles, either because of its high impact in producing changes or because they are currently ignored. Finally, boxes with a low number of strategies (L) are areas that need to be addressed, but where the gaps are smaller. Both, H and blank boxes can be interpreted as areas in which gaps -from an implementation science approach- still persist, because either they have been explicitly identified as relevant in dealing with the global health problems selected or because have been ignored in the analysis.

The results presented (including the construction of sub-categories) should be considered as an example of applying the synthesis to a particular set of information. Consequently, even though the synthesis can be generalized to other contexts, the results derived from its application to the Lancet paper may not. The example intends to show how the structure presented in Fig. 2 is useful to capture implementation strategies in different settings and to organize implementation priorities.

## Discussion

The aim of this paper is to provide a starting point for practitioners and decision makers to the field of implementation science, by compiling and classifying articles and concepts, synthesizing the existing implementation science frameworks in health care into a simplified scheme, and illustrating its use to identify current challenges in the implementation of priority programs in global health. The initial motivation behind it was to review the existence of several frameworks in the field that identify several success and failure factors, and to describe detailed implementation strategies. This information generates a trade-off, particularly for practitioners: on the one hand, more information and tools are available but, on the other hand, it generates confusion, hindering the selection of a model for implementing interventions in the practice.

We constructed a synthesis of frameworks used in implementation science to reduce the complexity in the field and offer guidance to policy makers and practitioners. The synthesis takes into account components of different types of models, capturing the wide variety of approaches emerging in the field. With this in mind, the proposed framework is not intended to replace other (more detailed) models but to help practitioners and decision makers to select the proper model, by providing a panoramic view of different types of models and their elements. Its flexibility permits the revision of particular categories, adding new subcategories or going deep into existing ones, depending on the focus of the intervention; other frameworks can be used to complement the analysis if the intervention requires a particular focus, e.g. address a specific disease [55], a dealing with environmental factors [47] or aligning interventions with recipients [31, 57].

The approach has several limitations. First, we used a review that was, at least initially, focused on IS frameworks: it is possible that there are other (for example disease-specific) frameworks that may not have surfaced in our review. However, we were interested in broad, foundational frameworks. Additionally, the strategy of complementing the initial search with experts’ suggestions and the use of snowball sampling to include extra articles in the review help dealing with this issue. Second, the search strategy was a targeted and not a systematic review. This decision was based on the difficulty of performing a traditional systematic review in the field, as identified by the previous literature. However, the used search strategy allowed to identify a literature beyond IS frameworks and to build a broader network of IS-related works that enriched the analysis. Third, there is an unavoidable trade-off between specificity and simplicity. Simplicity is relevant for practitioners, but having a framework that can be applied to solve a particular problem too. The proposed summary seeks simplicity by merging information from several models, mixing time-based and component-based frameworks, into a single structure with six elements. In this sense, the synthesis should be considered as an initial step for implementing innovations and understanding implementation gaps. Fourth, the application of the framework using the Lancet series produces ad-hoc results that are not necessarily applicable to other context. Different results –including the definition of sub-categories and identification of gaps- would have arisen by using different sources of data. Nevertheless, the utilization of the Lancet series must be seen as an example to illustrate one way in which IS and the proposed framework can be used to identify priority areas for intervention in global health.

## Conclusions

The aim of this article was synthesize and analyze key implementation science frameworks in health care, in order to develop a tool to identify core challenges in the implementation of priority programs in global health. The analysis reveals the presence of several models and frameworks in implementation science, including different definitions and approaches.

The process of compelling, classifying and comparing different frameworks uncovered the huge diversity of models but also highlighted common elements that can be condensed in a general simplified tool. The analysis also showed the usefulness of applying an implementation science approach to identify and prioritize gaps in implementation strategies in global health.

We hope this synthesis can be a useful tool for policy makers, particularly in identifying the relevant elements and phases in an implementation process, and as an instrument to prioritize areas for action. The synthesis can only be improved by using and adapting it to different settings and implementation challenges. We encourage policy makers to use and test the proposed synthesis in designing, implementing and evaluating implementation initiatives in applied health care programs.

## Additional files

Additional file 1: List of articles reviewed (DOCX 88 kb)

Additional file 2: List of frameworks reviewed (DOCX 113 kb)

## Data Availability

The datasets used and/or analyzed during the current study are available from the corresponding Availability of data and author on reasonable request.

## Abbreviations

HIV/AIDS: Human Immunodeficiency Virus/ Acquired Immunodeficiency Syndrome
IS: Implementation Science
M&CH: Maternal and Child Health
MDGs: Millennium Development Goals
NCDs: Non-Communicable Diseases
TB: Tuberculosis

## References

[1] Roberts ET, Matthews DD (2012). HIV and chemoprophylaxis, the importance of considering social structures alongside biomedical and behavioral intervention. Soc Sci Med.

[2] Damschroder LJ, Hagedorn HJ (2011). A guiding framework and approach for implementation research in substance use disorders treatment. Psychol Addict Behav.

[3] Steckler A, McLeroy KR (2008). The Importance of External Validity. Am J Public Health.

[4] Victora CG, Habitch JP, Bryce J (2004). Evidence-based public health: moving beyond randomized trials. Am J Public Health.

[5] Tabak RG, Khoong EC, Chambers DA, Brownson RC (2012). Bridging research and practice: models for dissemination and implementation research. Am J Prev Med.

[6] Moullin JC, Sabater-Hernández D, Fernandez-Llimos F, Benrimoj SI (2015). A systematic review of implementation frameworks of innovations in healthcare and resulting generic implementation framework. Health Res Policy Syst.

[7] Greenhalgh T, Robert G, Macfarlane F, Bate P, Kyriakidou O (2004). Diffusion of innovations in service organizations: systematic review and recommendations. Milkbank Quarterly.

[8] Eccles MP, Mittman BS (2006). Welcome to Implementation Science. Implement Sci.

[9] Michie S, Johnston M, Abraham C, Lawton R, Parker D, Walker A (2005). Making psychological theory useful for implementing evidence based practice: a consensus approach. Qual Saf Health Care.

[10] Colquhoun H, Leeman J, Michie S, Lokker C, Bragge P, Hempel S, McKibbon KA, Peters G-JY, Stevens KR, Wilson MG, Grimshaw J (2014). Towards a common terminology: a simplified framework of interventions to promote and integrate evidence into health practices, systems, and policies. Implement Sci.

[11] Kruk ME (2014). More Health for the Money - Toward a More Rigorous Implementation Science. Sci Transl Med.

[12] Davies P, Walker AE, Grimshaw JM (2010). A systematic review of the use of theory in the design of guideline dissemination and implementation strategies and interpretation of the results of rigorous evaluations. Implement Sci.

[13] Feldstein AC, Glasgow RE (2008). A practical, robust implementation and sustainability model (PRISM) for integrating research findings into practice. Jt Comm J Qual Patient Saf.

[14] Fixsen DL, Naoom SF, Blasé KA, Friedman RM, Wallace F (2005). Implementation Research: A Synthesis of the Literature.

[15] Spiegelman D (2016). Evaluating public health interventions: examples, definitions, and a personal note. Am J Public Health.

[16] Odeny TA, Padian N, Doherty MC, Baral S, Beyrer C, Ford N, Geng EH (2015). Definitions of implementation science in HIV/AIDS. Lancet..

[17] Durlak JA, DuPre EP (2008). Implementation matters: a review of research on the influence of implementation on program outcomes and the factors affecting implementation. Am J Community Psychol.

[18] Rabin BA, Brownson RC, Haire-Joshu D, Kreuter MW, Weaver NL (2008). A glossary for dissemination and implementation research in health. J Public Health Management Practice.

[19] Nilsen P (2015). Making sense of implementation theories, models and frameworks. Implement Sci.

[20] Chaudoir SR, Dugan AG, Barr CHI (2013). Measuring factors affecting implementation of health innovations: a systematic review of structural, organizational, provider, patient, and innovation level measures. Implement Sci.

[21] Damschroder LJ, Aron DC, Keith RE, Kirsh SR, Alexander JA, Lowery JC (2009). Fostering implementation of health services research findings into practice: a consolidated framework for advancing implementation science. Implement Sci.

[22] Rogers EM (2003). Diffusion of innovations.

[23] Grol R, Bosch MC, Hulscher ME, Eccles MP, Wensing M (2007). Planning and studying improvement in patient care: the use of theoretical perspectives. Milbank Q.

[24] May C (2013). Towards a general theory of implementation. Implement Sci.

[25] Fisher ES, Shortell SM, Savitz LA (2016). Implementation science: a potential catalyst for delivery system reform. JAMA..

[26] Pauwles L (2000). Taking the visual turn in research and scholarly communication. Vis Sociol.

[27] Ajzen I (1991). The theory of planned behavior. Organ Behav Hum Decis Process.

[28] Barker PM, Reid A, Schall MW (2016). A framework for scaling up health interventions: lessons from large-scale improvement initiatives in Africa. Implement Sci.

[29] Bero LA, Grilli R, Grimshaw RM, Harvey E, Oxman AD, Thomson MA (1998). Closing the gap between research and practice: an overview of systematic reviews of interventions to promote implementation of research findings by health care professionals. BMJ..

[30] Cane J, O’Connor D, Michie S (2012). Validation of the theoretical domains framework for use in behaviour change and implementation research. Implement Sci.

[31] Carroll C, Patterson M, Wood S, Booth A, Rick J, Balain S (2007). A conceptual framework for implementation fidelity. Implement Sci.

[32] Craig P, Dieppe P, Macintyre S, Michie S, Nazareth I, Petticrew M (2008). Developing and evaluating complex interventions: the new Medical Research Council guidance. BMJ..

[33] Eccles MP, Armstrong D, Baker R, Cleary K, Davies H, Davies S, Glasziou P, Ilott I, Kinmonth A-L, Leng G, Logan S, Marteau T, Michie S, Rogers H, Rycroft-Malone J, Sibbald B (2009). An implementation research agenda. Implement Sci.

[34] Ferlie EW, Shortell SM (2001). Improving the quality of health care in the United Kingdom and the United States: a framework for change. Milkbank Quarterly..

[35] French SD, Green SE, O’Connor DA, McKenzie JE, Francis JJ, Michie S, Buchbinder R, Schattner P, Spike N, Grimshaw JM (2012). Developing theory-informed behaviour change interventions to implement evidence into practice: a systematic approach using the theoretical domains framework. Implement Sci.

[36] Glasgow RE, Vogt TM, Boles SM (1999). Evaluating the public health impact of health promotion interventions: the RE-AIM framework. Am J Public Health.

[37] Glisson C, Schoenwald SK (2005). The ARC organizational and community intervention strategy for implementing evidence-based children’s mental health treatments. Ment Health Serv Res.

[38] Green LW, Kreuter MW (2005). Health program planning: an educational and ecological approach.

[39] Grimshaw JM, Thomas R, MacLennan G, Fraser C, Ramsay C, Vale L, Whitty P, Eccles M, Matowe L, Shirran L, Wensing M, Dijkstra R, Donaldson C (2004). Effectiveness and efficiency of guideline dissemination and implementation strategies. Health Technol Assess.

[40] Grol R, Jones R (2000). Twenty years of implementation research. Fam Pract.

[41] Kilbourne AM, Neuman MS, Pincus HA, Bauer MS, Stall R (2007). Implementing evidence-based interventions in health care: applications of the replicating effective programs framework. Implement Sci.

[42] Kitson A, Harvey G, McCormack B (1998). Enabling the implementation of evidence based practice: a conceptual framework. Quality in Health Care.

[43] Kitson A, Rycroft-Malone J, Harvey G, McCormack B, Seers K, Titchen A (2008). Evaluating the successful implementation of evidence into practice using the PARiHS framework: theoretical and practical challenges. Implement Sci.

[44] Klein KJ, Sorra JS (1996). The challenge of innovation implementation. Acad Manag Rev.

[45] Madon T, Hofman KJ, Kupfer L, Glass RI (2007). Public health. Implementation science. Science.

[46] McCormack B, McCarthy G, Wright J, Slater P, Coffey A (2009). Development and testing of the context assessment index (CAI). Worldviews Evid-Based Nurs.

[47] McCormack B, Kitson A, Harvey G, Rycroft-Malone J, Titchen A, Seers K (2002). Getting evidence into practice: the meaning of `context. J Adv Nurs.

[48] Meyers DC, Durlak JA, Wandersman A (2012). The quality implementation framework: a synthesis of critical steps in the implementation process. Am J Community Psychol.

[49] Oxman AD, Thomson MA, Davis DA (1995). No magic bullets: a systematic review of 102 trials of interventions to improve professional practice. CMAJ..

[50] Rycroft-Malone J (2004). The PARIHS framework—a framework for guiding the implementation of evidence-based practice. J Nurs Care Qual.

[51] Rycroft-Malone J, Kitson A, Harvey G, McCormack B, Seers K, Titchen A, Estabrooks C (2002). Ingredients for change: revisiting a conceptual framework. Qual Saf Health Care..

[52] Schein EH (2010). Organizational culture and leadership.

[53] Senge P (1990). The fifth discipline: the art and practice of the learning organization.

[54] Simpson DD (2002). A conceptual framework for transferring research to practice. J Subst Abus Treat.

[55] Sivaram S, Sanchez MA, Rimer BK, Samet JM, Glasgow RE (2014). Implementation science in Cancer prevention and control: a framework for research and programs in low- and middle-income countries. Cancer Epidemiol Biomark Prev.

[56] Stetler CB, Legro MW, Wallace CM, Bowman C, Guihan M, Hagedorn H, Kimmel B, Sharp ND, Smith JL (2006). The role of formative evaluation in implementation research and the QUERI experience. J Gen Intern Med.

[57] Stetler CB, McQueen L, Demakis J, Mittman B (2008). An organizational framework and strategic implementation for system-level change to enhance research-based practice: QUERI series. Implement Sci.

[58] Stetler CB, Damschroder LJ, Helfrich CD, Hagedorn HJ (2011). A guide for applying a revised version of the PARIHS framework for implementation. Implement Sci.

[59] Taylor MJ, McNicholas C, Nicolay C, Darzi A, Bell D, Reed JE (2014). Systematic review of the application of the plan–do–study–act method to improve quality in healthcare. BMJ Qual Saf.

[60] Victora CG, Requejo JH, Barros AJD, Berman P, Bhutta Z, Boerma T, Chopra M, de Francisco A, Daelmans B, Hazel E, Lawn J, Maliqi B, Newby H, Bryce J (2016). Countdown to 2015: a decade of tracking progress for maternal, newborn, and child survival. Lancet.

[61] Piot P, Karim SSA, Hecht R, Legido-Quigley H, Buse K, Stover J, Resch S, Ryckman T, Møgedal S, Dybul M, Goosby E, Watts C, Kilonzo N, McManus J, Sidibé M (2015). Defeating AIDS—advancing global health. Lancet..

[62] Ortblad KF, Salomon JA, Bärnighausen T, Atun R (2015). Stopping tuberculosis: a biosocial model for sustainable development. Lancet.

[63] Keshavjee S, Dowdy D, Swaminathan S (2015). Stopping the body count: a comprehensive approach to move towards zero tuberculosis deaths. Lancet..

[64] Furin J, Akugizibwe P, Ditiu L, Gray G, Palmero D, Zaidi S (2015). No one with HIV should die from tuberculosis. Lancet.

[65] Dickson KE, Simen-Kapeu A, Kinney MV, Huicho L, Vesel L, Lackritz E, de Graft JJ, von Xylander S, Rafi N, Sylla M, Mwansambo C, Daelmans B, Lawn JE (2014). Every newborn: health-systems bottlenecks and strategies to accelerate scale-up in countries. Lancet..

[66] Bonita R, Magnusson R, Bovet P, Zhao D, Malta DC, Geneau R, Suh I, Thankappan KR, McKee M, Hospedales J, de Courten M, Capewell S, Beaglehole R (2013). Country actions to meet UN commitments on non-communicable diseases: a stepwise approach. Lancet..

[67] Ruel MT, Alderman H (2013). Nutrition-sensitive interventions and programmes: how can they help to accelerate progress in improving maternal and child nutrition?. Lancet..

[68] Moonen B, Cohen JM, Snow RW, Slutsker L, Drakeley C, Smith DL, Abeyasinghe RR, Rodriguez MH, Maharaj R, Tanner M, Targett G (2010). Operational strategies to achieve and maintain malaria elimination. Lancet..

[69] Beyrer C, Malinowska-Sempruch K, Kamarulzaman A, Kazatchkine M, Sidibe M, Strathdee SA (2010). Time to act: a call for comprehensive responses to HIV in people who use drugs. Lancet..

[70] Morris SS, Cogill B, Uauy R (2008). Effective international action against undernutrition: why has it proven so difficult and what can be done to accelerate progress?. Lancet..

[71] Bhutta ZA, Ahmed T, Black RE, Cousens S, Dewey K, Giugliani E, Haider BA, Kirkwood B, Morris SS, Sachdev HP, Shekar M (2008). What works? Interventions for maternal and child undernutrition and survival. Lancet..

[72] Bryce J, Coitinho D, Darnton-Hill I, Pelletier D, Pinstrup-Andersen P (2008). Maternal and child undernutrition: effective action at national level. Lancet..

