# Supplementary material for: A synthesis of implementation science frameworks and application to global health gaps
Source: Glob Health Res Policy. 2019 Aug 27;4:25. doi: 10.1186/s41256-019-0115-1 (PMC6712702; doi:10.1186/s41256-019-0115-1)
Supplement: Supplementary file 1 — List of articles reviewed (DOCX 88 kb) [file 41256_2019_115_MOESM1_ESM.docx]

**Appendix 1: List of articles reviewed**

| **#** | **Short reference** | **Reference number** | **# citations (total)** | **# citations as reference framework** | **Includes framework?** | **Based on systematic search?** | **Includes classification? *(Articles, definitions, theories)*** |
| --- | --- | --- | --- | --- | --- | --- | --- |
| 1 | Ajzen (1991). The theory of planned behavior | 27 | 6 | 1 | Y (behavior) | N | N |
| 2 | Barker et al (2016). A framework for scaling up health interventions: lessons from large-scale improvement initiatives in Africa | 28 | 0 | 0 | Y | Y | N |
| 3 | Bero et al. (1998). Closing the gap between research and practice: an overview of systematic reviews of interventions to promote implementation of research findings by health care professionals | 29 | 6 | 0 | N | Y | Y |
| 4 | Cane et al (2012). Validation of the theoretical domains framework for use in behaviour change and implementation research | 30 | 2 | 0 | Y | N | N |
| 5 | Carroll et al (2007). A conceptual framework for implementation fidelity | 31 | 2 | 0 | Y | N | N |
| 6 | Chaudoir et al (2013). Measuring factors affecting implementation of health innovations: a systematic review of structural, organizational, provider, patient, and innovation level measures | 19 | 0 | 0 | Y | Y | Y |
| 7 | Craig et al (2008). Developing and evaluating complex interventions: the new Medical Research Council guidance | 32 | 3 | 0 | Y | N | N |
| 8 | Damschroder and Hagedorn (2011). A Guiding Framework and Approach for Implementation Research in Substance Use Disorders Treatment | 2 | 0 | 0 | Y | N | N |
| 9 | Damschroder et al. (2009). Fostering implementation of health services research findings into practice: A consolidated framework for advancing implementation science | 20 | 11 | 5 | Y | Y | N |
| 10 | Durlak and DuPre (2008). Implementation Matters: A Review of Research on the Influence of Implementation on Program Outcomes and the Factors Affecting Implementation | 16 | 4 | 3 | Y | Y | Y |
| 11 | Eccles and Mittman (2006). Welcome to implementation science | 8 | 6 | 0 | N | N/A | N |
| 12 | Eccles et al (2009). An implementation research agenda | 33 | 0 | 0 | N | N/A | N |
| 13 | Feldstein and Glasgow (2008). A practical, robust implementation and sustainability model (PRISM) for integrating research findings into practice | 12 | 5 | 4 | Y | Y | N |
| 14 | Ferlie and Shortell (2001). Improving the quality of health care in the United Kingdom and the United States: a framework for change | 34 | 6 | 1 | Y | N | Y |
| 15 | Fixsen et al. (2005). Implementation research: A synthesis of the literature | 13 | 10 | 3 | Y | Y | N |
| 16 | Fischer et al (2016). Implementation Science: A Potential Catalyst for Delivery System Reform | 24 | 0 | 0 | Y | N | N |
| 17 | French et al (2012). Developing theory-informed behaviour change interventions to implement evidence into practice: a systematic approach using the Theoretical Domains Framework | 35 | 1 | 0 | Y | N | N |
| 18 | Glasgow et al. (1999). Evaluating the public health impact of health promotion interventions: The RE-AIM framework | 36 | 7 | 0 | Y | N | N |
| 19 | Glisson and Schoenwald (2005). The ARC organizational and community intervention strategy for implementing evidence-based children’s mental health treatments | 37 | 4 | 4 | Y | N | N |
| 20 | Green and Kreuter (2005). Health Program Planning: An Educational and Ecological Approach | 38 | 7 | 3 | Y | N | N |
| 21 | Greenhalgh el al. (2004). Diffusion of innovations in service organizations: Systematic review and recommendations | 7 | 15 | 6 | Y | Y | Y |
| 22 | Grimshaw et al. (2004). Effectiveness and efficiency of guideline dissemination and implementation strategies | 39 | 6 | 0 | N | Y | N |
| 23 | Grol and Jones (2000). Twenty years of implementation research | 40 | 1 | 0 | N | N/A | N |
| 24 | Grol et al. (2007). Planning and studying improvement in patient care: The use of theoretical perspectives | 22 | 6 | 1 | N | Y | Y |
| 25 | Kilbourne et al. (2007). Implementing evidence-based interventions in health care: Applications of the replicating effective programs framework | 41 | 4 | 4 | Y | Y | N |
| 26 | Kitson et al. (1998). Enabling the implementation of evidence based practice: a conceptual framework | 42 | 11 | 4 | Y | N | N |
| 27 | Kitson et al. (2008). Evaluating the successful implementation of evidence into practice using the PARiHS framework | 43 | 6 | 4 | N | N/A | N |
| 28 | Klein and Sorra (1996). The challenge of innovation implementation | 44 | 8 | 4 | Y | N | N |
| 29 | Madon et al (2007). Implementation Science | 45 | 2 | 0 | N | N/A | N |
| 30 | May (2013). Towards a general theory of implementation | 23 | 2 | 1 | Y | N | N |
| 31 | McCormack et al. (2009). Development and Testing of the Context Assessment Index (CAI) | 46 | 3 | 1 | Y | N | N |
| 32 | McCormack et al. (2002). Getting evidence into practice: the meaning of 'context' | 47 | 7 | 2 | N | Y | N |
| 33 | Meyers et al. (2012). The Quality Implementation Framework: A Synthesis of Critical Steps in the Implementation Process | 48 | 2 | 1 | Y | Y | Y |
| 34 | Michie et al. (2005). Making psychological theory useful for implementing evidence based practice: a consensus approach | 25 | 6 | 0 | Y | N | Y |
| 35 | Moullin et al (2015). A systematic review of implementation frameworks of innovations in healthcare and resulting generic implementation framework | 6 | 0 | 0 | Y | Y | Y |
| 36 | Nilsen (2015). Making sense of implementation theories, models and frameworks | 18 | 0 | 0 | N | N/A | Y |
| 37 | Odeny et al (2015). Definitions of implementation science in HIV/AIDS | 15 | 0 | 0 | N | N/A | Y |
| 38 | Oxman et al (1995). No magic bullets: a systematic review of 102 trials of interventions to improve professional practice | 49 | 6 | 0 | N | Y | Y |
| 39 | Rabin et al (2008). A Glossary for Dissemination and Implementation Research in Health | 17 | 5 | 0 | N | N/A | N |
| 40 | Rogers (2003). Diffusion of innovations | 21 | 28 | 7 | Y | N | Y |
| 41 | Rycroft-Malone (2004). The PARIHS Framework - A Framework for Guiding the Implementation of Evidence-based Practice | 50 | 5 | 2 | Y | N | N |
| 42 | Rycroft-Malone et al. (2002). Ingredients for change: revisiting a conceptual framework | 51 | 7 | 2 | Y | N | N |
| 43 | Schein (2010). Organizational culture and leadership | 52 | 7 | 0 | Y (organizational culture) | N | N |
| 44 | Senge (1990). The Fifth Discipline: The Art and Practice of the Learning Organization. | 53 | 7 | 0 | N | N | Y |
| 45 | Simpson (2002). A conceptual framework for transferring research to practice | 54 | 5 | 1 | Y | N | N |
| 46 | Sivaram et al (2014). Implementation Science in Cancer Prevention and Control: A Framework for Research and Programs in Low- and Middle-Income Countries | 55 | 0 | 0 | Y | N | N |
| 47 | Spiegelman (2015). Evaluating Public Health Interventions: Examples, Definitions, and a Personal Note | 14 | 0 | 0 | N | N/A | N |
| 48 | Stetler et al (2006). The Role of Formative Evaluation in Implementation Research and the QUERI Experience | 56 | 6 | 0 | N | N/A | N |
| 49 | Stetler et al (2008). An organizational framework and strategic implementation for system-level change to enhance research-based practice: QUERI Series | 57 | 2 | 1 | N | N/A | N |
| 50 | Stetler et al (2011). A Guide for applying a revised version of the PARIHS framework for implementation | 58 | 1 | 1 | Y | N | N |
| 51 | Tabak et al (2012). Bridging Research and Practice Models for Dissemination and Implementation Research | 5 | 3 | 0 | N | N/A | Y |
| 52 | Taylor et al (2014). Systematic review of the application of the plan–do–study–act method to improve quality in healthcare | 59 | 0 | 0 | Y | Y | Y |
