# Supplementary material for: A synthesis of implementation science frameworks and application to global health gaps
Source: Glob Health Res Policy. 2019 Aug 27;4:25. doi: 10.1186/s41256-019-0115-1 (PMC6712702; doi:10.1186/s41256-019-0115-1)
Supplement: Supplementary file 2 — List of frameworks reviewed (DOCX 113 kb) [file 41256_2019_115_MOESM2_ESM.docx]

**Appendix 2: List of frameworks reviewed**

| **#** | **Short reference** | **Reference number** | **Associated framework** | **Focus** | **Context** |
| --- | --- | --- | --- | --- | --- |
| 1 | Barker et al. (2016). A framework for scaling up health interventions: lessons from large-scale improvement initiatives in Africa | 28 | Framework for Going to Full Scale | Time + Components | Africa |
| 2 | Cane and Michie (2012). Validation of the theoretical domains framework for use in behaviour change and implementation research | 30 | Theoretical Domains Framework (TDF) | Components | General |
| 3 | Carroll et al. (2007). A conceptual framework for implementation fidelity | 31 | Implementation fidelity | Components | General |
| 4 | Chaudoir et al. (2013). Measuring factors affecting implementation of health innovations: a systematic review of structural, organizational, provider, patient, and innovation level measures | 19 | Structural, organizational, provider, patient, and innovation level measures | Components | General |
| 5 | Craig et al. (2008). Developing and evaluating complex interventions: the new Medical Research Council guidance | 32 | Evaluation of complex interventions | Time | General |
| 6 | Damschroder and Hagedorn (2011). A Guiding Framework and Approach for Implementation Research in Substance Use Disorders Treatment. | 2 | Consolidated Framework for Implementation Research (CFIR) | Components | Substance use disorder treatment |
| 7 | Damschroder et al. (2009). Fostering implementation of health services research findings into practice: a consolidated framework for advancing implementation science | 20 | Consolidated Framework for Implementation Research (CFIR) | Components | General |
| 8 | Durlak and DuPre (2008). Implementation Matters: A Review of Research on the Influence of Implementation on Program Outcomes and the Factors Affecting Implementation | 16 | Interactive System Framework (ISF) | Components | Children and adolescences |
| 9 | Feldstein and Glasgow (2008). A Practical, Robust Implementation and Sustainability Model (PRISM) for Integrating Research Findings into Practice | 12 | Practical, Robust Implementation and Sustainability Model (PRISM) | Components + Time | General |
| 10 | Ferlie and Shortell (2001). Improving the quality of health care in the United Kingdom and the United States: a framework for change | 34 | Quality improvement | Components | General |
| 11 | Fisher et al. (2016). Implementation Science: A Potential Catalyst for Delivery System Reform | 24 | Consolidated Framework for Implementation Research (CFIR) | Components | General |
| 12 | Fixsen et al. (2005). Implementation Research: A Synthesis of the Literature | 13 | Conceptual Framework for Implementation of Defined Practices and Programs | Components + Time | General |
| 13 | French et al. (2012). Developing theory-informed behaviour change interventions to implement evidence into practice: a systematic approach using the Theoretical Domains Framework | 35 | Theoretical Domains Framework (TDF) | Components | Acute low back pain in primary care |
| 14 | Glasgow et al. (1999). Evaluating the Public Health Impact of Health Promotion Interventions: The RE-AIM Framework | 36 | RE-AIM | Components + Time | Public health promotion |
| 15 | Glisson and Schoenwald (2005). The ARC Organizational and Community Intervention Strategy for Implementing Evidence-Based Children’s Mental Health Treatments | 37 | Availability, Responsiveness and Continuity (ARC) | Components + Time | Mental health, Community-based settings |
| 16 | Green and Kreuter (2005). Health Program Planning: An Educational and Ecological Approach | 38 | Precede-Proceed Model | Time + Components | General |
| 17 | Greenhalgh et al. (2004). Diffusion of innovations in service organizations: systematic review and recommendations | 7 | Diffusion of innovations | Components | Service organizations |
| 18 | Grol and Jones (2000). Twenty years of implementation research | 40 |  | Time + Components | General |
| 19 | Kilbourne et al. (2007). Implementing evidence-based interventions in health care: application of the replicating effective programs framework | 41 | Replicating Effective Programs (REP) | Time | Community-based settings |
| 20 | Kitson et al. (1998). Enabling the implementation of evidence based practice: a conceptual framework | 42 | Promoting Action on Research Implementation in Health Services (PARIHS) | Components | General |
| 21 | Klein and Sorra (1996). The Challenge of Innovation Implementation | 44 | Innovation effectiveness | Components | General |
| 22 | May (2013). Towards a general theory of implementation | 23 | General theory of implementation | Components | Nursing clinical guidelines |
| 23 | McCormack et al. (2009). Development and Testing of the Context Assessment Index (CAI) | 46 | Promoting Action on Research Implementation in Health Services (PARIHS) | Components | General |
| 24 | McCormack et al. (2002). Getting evidence into practice: the meaning of `context' | 47 | Promoting Action on Research Implementation in Health Services (PARIHS) | Components | General |
| 25 | Meyers et al. (2012). The Quality Implementation Framework: A Synthesis of Critical Steps in the Implementation Process | 48 | Quality Implementation Framework (QIF) | Time | General |
| 26 | Michie et al. (2005). Making psychological theory useful for implementing evidence based practice: a consensus approach | 25 | Behavior change | Components | General |
| 27 | Moullin et al. (2015). A systematic review of implementation frameworks of innovations in healthcare and resulting generic implementation framework | 6 | Generic Implementation Framework | Time + Components | General |
| 28 | Rabin et al. (2008). A Glossary for Dissemination and Implementation Research in Health | 17 |  | Components | General |
| 29 | Rogers (2003). Diffusion of innovations | 21 | Diffusion of innovations | Components + Time | General |
| 30 | Rycroft-Malone (2004). The PARIHS Framework—A Framework for Guiding the Implementation of Evidence-based Practice | 50 | Promoting Action on Research Implementation in Health Services (PARIHS) | Components | General |
| 31 | Rycroft-Malone et al. (2002). Ingredients for change: revisiting a conceptual framework | 51 | Promoting Action on Research Implementation in Health Services (PARIHS) | Components | General |
| 32 | Sivaram et al. (2014). Implementation Science in Cancer Prevention and Control: A Framework for Research and Programs in Low- and Middle-Income Countries | 55 |  | Time | Cancer control/ Low and middle-income countries |
| 33 | Simpson (2002). A conceptual framework for transferring research to practice | 54 | Technology transfer | Components | Community drug treatment |
| 34 | Stetler et al. (2011). A Guide for applying a revised version of the PARIHS framework for implementation | 58 | Promoting Action on Research Implementation in Health Services (PARIHS) | Components | General |
| 35 | Taylor et al. (2014). Systematic review of the application of the plan–do–study–act method to improve quality in healthcare | 59 | Plan-Do-Study-Act (PDSA) | Time | General |
